# Risk factors for alcohol-related injury experienced within a year: results of a nationwide large-scale internet survey

**DOI:** 10.1016/j.abrep.2026.100735

**Published:** 2026-08-12

**Authors:** Kazuya Okushin, Takahiro Tabuchi, Takashi Yoshioka, Tomohiro Tanaka, Takuma Kaneko, Keisuke Mabuchi, Yuki Matsushita, Tomoharu Yamada, Takuma Nakatsuka, Tatsuya Minami, Masaya Sato, Yotaro Kudo, Kazuhiko Koike, Mitsuhiro Fujishiro, Ryosuke Tateishi

**Affiliations:** a Department of Gastroenterology, Graduate School of Medicine, The University of Tokyo, Tokyo, Japan; b Department of Infection Control and Prevention, Graduate School of Medicine, The University of Tokyo, Tokyo, Japan; c Division of Epidemiology, School of Public Health, Tohoku University Graduate School of Medicine, Japan; d Institute of Clinical Epidemiology, Showa Medical University, Japan; e Shizuoka Graduate University of Public Health, Shizuoka, Japan; f Division of Gastroenterology and Hepatology, University of Iowa Carver College of Medicine, Iowa City, IA, USA; g Department of Health Management and Policy, College of Public Health, University of Iowa, Iowa City, IA, USA; h Kanto Central Hospital, Tokyo, Japan

**Keywords:** Alcohol-by-volume, Ready-to-drink alcoholic beverage, Alcohol-related self-injury, Alcohol-related harm to others, AUDIT, Type of alcoholic beverage

## Abstract

**Background:**

Alcohol-related injuries, including self-injury and harm to others, have multifactorial causes other than alcohol consumption amount and frequency. Here we investigated the determinants of alcohol-related self-injury and harm to others by focusing on the characteristics of alcoholic beverages.

**Methods:**

We analyzed data from a nationwide internet-based cohort survey conducted in Japan in 2024–2025. Among 11,443 continuous drinkers, alcohol-related self-injury and harm to others within the past year were assessed. Weighted logistic regression models using inverse probability weighting were used to estimate adjusted odds ratios (aORs) and 95% confidence intervals (CIs) with adjustment for demographic, socioeconomic, and clinical characteristics, drinking behaviors, and beverage types.

**Results:**

Of the study population, 285 and 122 participants experienced alcohol-related self-injury and harm to others, respectively. Younger age and frequent heavy episodic drinking showed strong dose–response associations with self-injury and harm to others. Diabetes was associated with increased risk, and depression showed a positive but not consistently statistically significant association. Wine consumption was negatively associated with self-injury (aOR, 0.40; 95% CI, 0.24–0.65; *p* < 0.001), whereas beer and moderate alcohol-by-volume (4–7%) ready-to-drink beverages were negatively associated with harm to others (beer: aOR, 0.38; 95% CI, 0.15–0.96; *p* = 0.04; 4–7% *chū-hai*: aOR, 0.29; 95% CI, 0.13–0.63; *p* = 0.002).

**Conclusions:**

Alcohol-related self-injury and harm to others share common and distinct determinants across demographic, clinical, behavioral, and beverage-related factors. These findings suggest that alcohol-related injury risk is primarily driven by drinking pattern and intensity, while beverage-specific associations may provide exploratory insights.

## Highlights

• Alcohol-related self-injury and harm to others share common and distinct determinants.
• Younger age and higher frequency of heavy episodic drinking showed consistent positive associations with injury outcomes.
• Beverage characteristics exhibited heterogeneity in their associations with injury outcomes, providing exploratory insights.

## 1 Introduction

Alcohol consumption is a major contributor to the global injury burden and responsible for significant trauma-related morbidity and mortality (Cherpitel, Ye, Bond, Room, & Borges, 2012; Chikritzhs & Livingston, 2021). Acute intoxication impairs an individual's psychomotor performance, balance, and judgment, thereby increasing the likelihood of unintentional injuries such as road traffic crashes, falls, burns, and drowning (Rehm et al., 2009). Alcohol also plays a pivotal role in intentional injury. Excessive drinking increases the risk of interpersonal violence, including intimate partner violence, and contributes substantially to self-inflicted harm, including suicidal behaviors (Borges, Walters, & Kessler, 2000; Foran & O'Leary, 2008).

Epidemiological studies have consistently demonstrated a clear dose–response relationship between alcohol consumption volume and the risk of any injury (Cherpitel, Ye, Bond, Borges, & Monteiro, 2015; Kruckow et al., 2023; Taylor et al., 2010). Frequent and episodic heavy drinking reportedly predicts any injury, including self-harm and interpersonal violence (Cherpitel, Witbrodt, Ye, & Korcha, 2018). The increased risk of injury is also seen at low levels of drinking regardless of the presence of alcohol dependence (Borges et al., 2006).

Despite robust evidence linking alcohol quantity and drinking patterns with injury risk, the influence of alcoholic beverage types on injury outcomes has not been well elucidated. Flavored alcoholic beverages (FABs)—including malt-based drinks, premixed cocktails, and supersized alcopops—have been implicated in risky drinking behaviors and adverse consequences, particularly among underaged and young adult populations (Albers et al., 2015; Fortunato et al., 2014; Metzner & Kraus, 2008). In Japan, one representative FAB is *chū-hai*, a ready-to-drink (RTD) fruit-based cocktail typically containing 4–7% alcohol-by-volume (ABV). Since 2009, the introduction of “strong *chū-hai*” products with 8–9% ABV has dramatically expanded their popularity. These beverages share the following characteristics with supersized alcopops in Western countries: high alcohol concentration, low cost, and ease of consumption (Rossheim, Thombs, & Treffers, 2018). Consequently, concerns have been raised about their contribution to alcohol-related injuries.

An individual's drinking habits are closely associated with depression and lifestyle-related diseases including diabetes (Conner, Pinquart, & Gamble, 2009; Rehm et al., 2009). These comorbid conditions independently increase the risk of both self-injury and harm to others (Fan et al., 2024; Fazel et al., 2015; Wang, An, Shi, & Zhang, 2017; Wu, Zhang, Wang, & Huang, 2024), suggesting that harmful drinking may act synergistically with underlying mental or physical health vulnerabilities. Therefore, this study aimed to identify the risk factors, including alcohol drinking behaviors, associated with self- and other-directed harm in the general population.

## 2 Methods

### 2.1 Design

This study used data from the Japan Society and New Tobacco Internet Survey (JASTIS), a longitudinal study project comprising a series of large-scale annual online surveys undertaken since 2015. As of June 1, 2025, nine survey waves have been conducted. In this longitudinal study, the present analysis was designed as a prospective cohort study using data from two consecutive survey waves, the 2024 JASTIS (*n* = 32,000) and 2025 JASTIS (*n* = 28,000) surveys conducted from January to February each year by Rakuten Insight, Inc. (Tokyo, Japan), to examine alcohol-related injury outcomes at follow-up among continuous drinkers. The details of JASTIS were published previously (Tabuchi, Shinozaki, Kunugita, Nakamura, & Tsuji, 2019). All questionnaires were designed such that the respondents had to answer each question before proceeding to the next, thereby ensuring that the missing data were not generated.

### 2.2 Participants

Among the respondents of the 2024 JASTIS (32,000) and 2025 JASTIS (28,000), we excluded those for whom data were inadequate, such as a short response time (<600 s), failure to answer an attention check question, reporting all listed drug use categories, listing all health conditions, or reporting more than 15 family members. We further excluded current non-drinkers defined as participants who answered “Never” to the question “How often do you have a drink containing alcohol?” (Alcohol Use Disorders Identification Test [AUDIT] Q1) (Saunders, Aasland, Babor, de la Fuente, & Grant, 1993). We then identified eligible respondents for both surveys.

### 2.3 Questions related to alcoholic usage

We further asked respondents the questions “How many drinks containing alcohol do you have on a typical day when you are drinking?” (AUDIT Q2) and “How often do you have six or more drinks on one occasion?” (AUDIT Q3).

The use of each alcohol-containing drink was defined on the basis of the response to a specific question that we developed: “How often do you drink the following alcohol-containing drinks” to which the response options were “Never,” “At least once in the past,” “Habitually in the past,” “Occasionally,” and “Almost every day.” Alcohol-containing drinks were divided into beer, Japanese-sake, sho-chū, wine, fruit wine, whiskey, *chū-hai*, and others. *Chū-hai* was further classified by the ABV (<1%, 3%, 4–7%, 8–9%). In this study, we defined those who responded “Occasionally,” and “Almost every day” as these categories reflect ongoing drinking behavior during the exposure period relevant to the follow-up outcomes. Respondents who selected “At least once in the past” or “Habitually in the past” were considered past users and were not classified as current users, as these categories likely represent historical exposure rather than current drinking patterns associated with injury risk during the follow-up period.

### 2.4 Other variables of interest

Next we examined each participant's current status of depression and diabetes mellitus (Borges, Cherpitel, Medina-Mora, & Mondragon, 2004; Fan et al., 2024; Fazel et al., 2015; Wang et al., 2017; Wu et al., 2024). We defined these diseases as “present” when the respondents reported current complications for each disease. The following variables were examined: anthropometric parameters (Hingson, Heeren, Jamanka, & Howland, 2000; Hingson, Heeren, & Winter, 2006), smoking status (Grant, Hasin, Chou, Stinson, & Dawson, 2004), educational level, and household income (Collins, 2016).

### 2.5 Outcomes

Alcohol-related injuries at 1 year of follow-up was the primary study outcome. In the 2025 JASTIS, specific questionnaires following the AUDIT were set as follows: “Have you ever injured yourself as a result of your drinking?” and “Has anyone else ever been injured because of your drinking?” The response options were “Never,” “Yes, but not in the past year,” and “Yes, in the past year.” We set those who responded “Yes, in the past year” as experiencing self-reported alcohol-related self-injury and alcohol-related harm to others within a year, respectively. These outcomes were based on participants' self-reported attribution of injury to their drinking.

### 2.6 Statistical analysis

As all of the JASTIS participants were panelists at a web agency and had access to the internet, our study may comprise a specific population with characteristics that differ from those of the general public in Japan. To determine the estimates of the general Japanese population with regard to the JASTIS respondents, we used an inverse probability weighting method that used propensity scores adjusted for the differences between the respondents of the online survey and the general public in Japan (Tabuchi et al., 2016; Yoshioka et al., 2023). To calculate the propensity scores, we combined the two datasets of the 2025 JASTIS and an external dataset that represented the general population in Japan. Of the combined dataset, we constructed a logistic regression model that regressed a binary categorical variable from the two datasets on the indicator variables of demographic and socioeconomic status. For the weights, we used samples from the 2019 Comprehensive Survey of Living Conditions, a nationally representative survey in Japan. The calculated propensity scores represent the probability of being a respondent in an internet survey.

The AUDIT-C score was calculated as the sum of AUDIT Q1–3 (range 0–12)(Bush, Kivlahan, McDonell, Fihn, & Bradley, 1998). Based on validation studies in Japan, hazardous drinking was defined as an AUDIT-C score ≥ 5 for men and ≥4 for women (Osaki et al., 2014).

Descriptive variables are expressed as numbers, proportions (%), weighted proportions, and 95% confidence intervals (CIs) unless otherwise specified. We used the chi-squared test to analyze categorical variables and Student's *t*-test to examine continuous variables.

We used multivariable logistic regression models to estimate adjusted odds ratios (aORs) and 95% CIs for alcohol-related self-injury and harm to others. Both models included age (Hingson et al., 2000; Hingson et al., 2006), sex, body mass index (BMI), educational attainment, low income (Collins, 2016), smoking status (never, past, or current) (Grant et al., 2004), the presence of depression (Koob & Volkow, 2016), the presence of diabetes, AUDIT Q1–3 (drinking frequency, usual drinking quantity, and heavy episodic drinking), and the current use of each alcoholic beverage as covariates. Age and BMI were included as continuous variables to reduce the number of covariates considering the number of events (Vittinghoff & McCulloch, 2007). Given the number of outcome events, model stability was evaluated by considering the event per variable and examining multicollinearity among predictors using variance inflation factors. Statistical significance was set at *p* < 0.05. All tests were two-tailed. All statistical analyses were performed using R software version 4.3 (R Foundation, Vienna, Austria; http://www.r-project.org/).

### 2.7 Ethical considerations

All participants responded to the online questionnaires after agreeing to provide online informed consent and confirming their intention to participate. A credit point known as “Epoints,” which could be used for online shopping and cash conversion, was provided to the participants as an incentive. All procedures were conducted in accordance with the ethical standards of the 1975 Declaration of Helsinki (revised in 2013). This study represents a secondary analysis of the JASTIS, and the present analysis used de-identified data without personal identifiers. This study was approved by the Institutional Review Board of Osaka International Cancer Institute (approval no. 20084), the Ethics Committee of Tohoku University Graduate School of Medicine (approval no. 2024–1-231), and the Research Ethics Committee of the Faculty of Medicine, the University of Tokyo (approval no. 2020336NI). The study protocol followed the STROBE guidelines for cohort studies.

## 3 Results

### 3.1 Participant recruitment

**Fig. 1:**
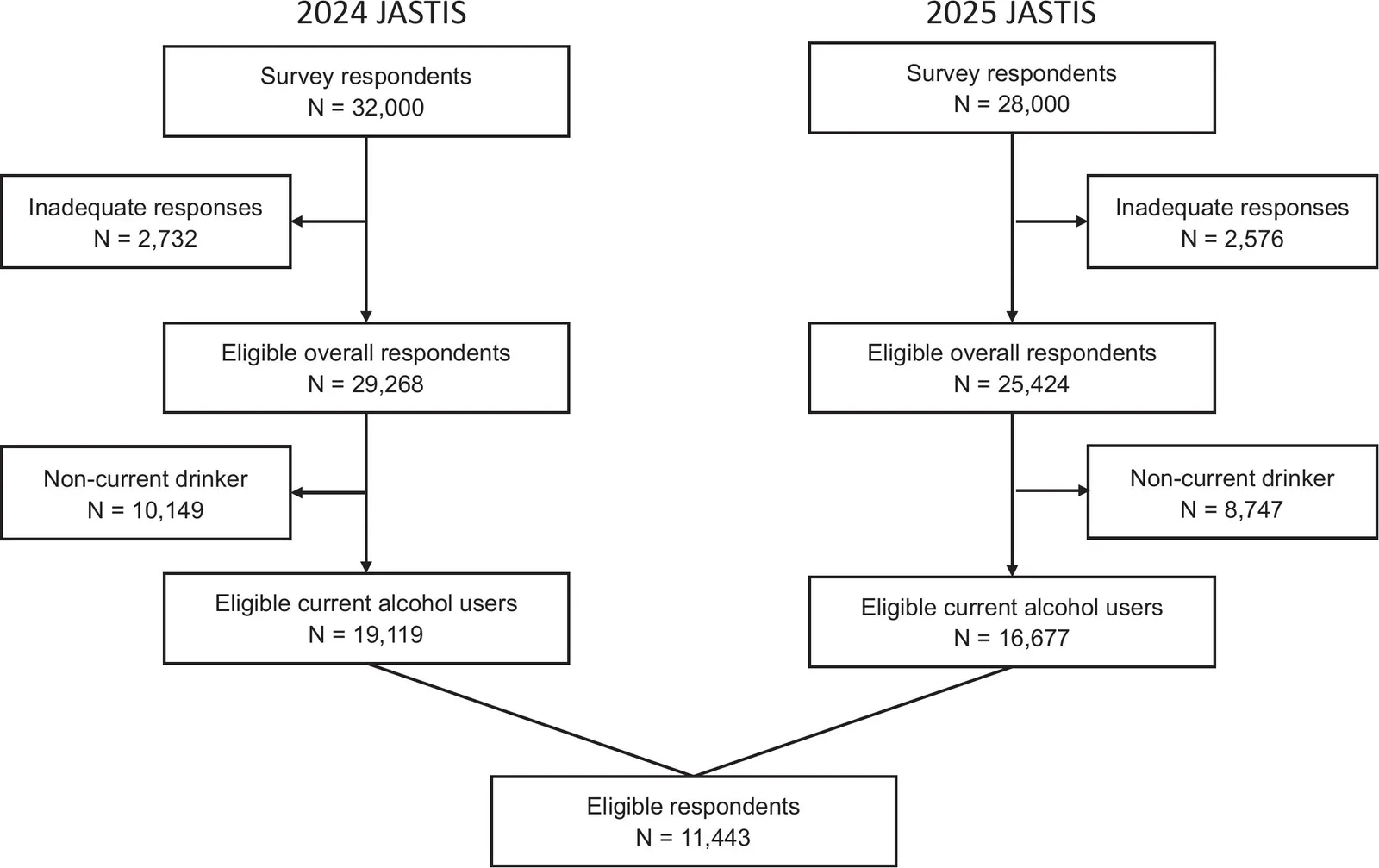
Patient recruitment flowchart.

Among the 32,000 and 28,000 respondents for the 2024 and 2025 JASTIS, 2732 and 2576 were excluded due to inadequate responses, respectively. We further excluded 10,149 and 8747 individuals who did not currently drink in 2024 and 2025, respectively. Finally, we extracted the data of 11,443 respondents from the 2024 and 2025 JASTIS identified as continuous drinkers from 2024 to 2025 for the analysis (Fig. 1).

### 3.2 Background characteristics

Among the eligible respondents, 59.5% were male, and age (median, 52.0 years [interquartile range (IQR), 38.0–65.0]) and BMI (median, 21.9 kg/m^2^ [IQR, 19.8–24.2]) were well distributed in the general population (Table 1). Although a college or higher degree was obtained by 53.8% of the respondents, the percentage with a low household income (<400 M yen/year) was 34.2%. Concerning comorbidities, depression and diabetes were diagnosed in 3.9% and 7.0% of patients, respectively. Answers to questions about alcohol consumption frequency and amount, namely AUDIT Q1–3, were well distributed in the general population. Regarding the current use of alcoholic drinks, beer was the most popular beverage, reported by 66.0% of the respondents. The AUDIT-C score was calculated as the sum of AUDIT Q1–3. The median AUDIT-C score was 3.0 (IQR 2.0–5.0). The distribution of AUDIT-C score categories was as follows: 35.6% for scores 0–2, 33.4% for scores 3–4, 21.9% for scores 5–7, and 9.1% for scores 8–12. Using validated cutoffs for hazardous drinking (AUDIT-C ≥ 5 for men and ≥4 for women), 38.2% were positive for hazardous drinking.

**Table 1:** Background characteristics of the study participants (*N* = 11,443).

Characteristics	n (%)	Weighted % (95% CI)
Age, years		
15–29	1497 (13.1)	13.0 (11.9–14.2)
30–39	1594 (13.9)	14.6 (13.6–15.7)
40–49	2067 (18.1)	18.7 (17.6–19.9)
50–59	2198 (19.2)	19.5 (18.3–20.6)
60–69	2194 (19.2)	18.2 (17.0–19.4)
70 and above	1893 (16.5)	15.9 (14.7–17.1)
Sex, male	6803 (59.5)	60.7 (59.2–62.2)
BMI, kg/m^2^		
<18.5	1352 (11.8)	10.7 (9.7–11.6)
18.5–25.0	7928 (69.3)	68.3 (66.9–69.8)
25.0–35.0	2087 (18.2)	20.3 (19.0–21.6)
35 and above	76 (0.7)	0.7 (0.5–0.9)
Educational attainment, College graduate or higher degree-holder	6156 (53.8)	29.1 (28.0–30.3)
Low income (<400 M yen)	3918 (34.2)	36.0 (34.5–37.5)
Smoking status		
Never	5955 (52.0)	47.7 (46.2–49.2)
Former	2964 (25.9)	28.6 (27.1–30.0)
Current	2524 (22.1)	23.7 (22.4–25.0)
Depression, present	449 (3.9)	3.8 (3.2–4.4)
Diabetes, present	806 (7.0)	7.8 (6.9–8.8)
Drinking frequency of alcohol containing drinks (AUDIT 1)		
Monthly or less	2984 (26.1)	26.0 (24.6–27.3)
2 to 4 times a month	2540 (22.2)	21.3 (20.1–22.5)
2 to 3 times a week	2079 (18.2)	17.7 (16.5–18.9)
4 or more times a week	3840 (33.6)	35.1 (33.6–36.5)
Drinking amount of alcohol containing drinks (AUDIT 2)		
1 or 2 drinks	7260 (63.4)	63.7 (62.2–65.2)
3 or 4 drinks	2676 (23.4)	22.8 (21.5–24.1)
5 or 6 drinks	1008 (8.8)	9.0 (8.1–9.8)
7, 8, or 9 drinks	297 (2.6)	2.7 (2.1–3.2)
10 or more drinks	202 (1.8)	1.9 (1.4–2.3)
Frequency of six or more drinks (AUDIT 3)		
Never	7285 (63.7)	64.7 (63.2–66.1)
Less than monthly	2222 (19.4)	18.1 (17.0–19.3)
Monthly	931 (8.1)	8.2 (7.4–9.0)
Weekly	631 (5.5)	5.2 (4.6–5.9)
Daily or almost daily	374 (3.3)	3.8 (3.1–4.5)
AUDIT-C score		
0 to 2	4075 (35.6)	35.3 (33.8–36.7)
3 to 4	3823 (33.4)	33.4 (31.9–34.8)
5 to 7	2507 (21.9)	22.0 (20.7–23.2)
8 to 12	1038 (9.1)	9.4 (8.5–10.3)
Hazardous drinking, present	4366 (38.2)	38.0 (36.6–39.5)
Current use of each alcohol containing beverages, yes		
Beer	7550 (66.0)	64.9 (63.4–66.4)
Chū-hai (<1%)	2383 (20.8)	21.0 (19.7–22.2)
Chū-hai (3%)	3598 (31.4)	31.3 (29.9–32.7)
Chū-hai (4–7%)	4745 (41.5)	41.3 (39.8–42.8)
Chū-hai (8–9%)	2894 (25.3)	25.8 (24.5–27.2)
Japanese Sake	4361 (38.1)	34.4 (33.0–35.9)
Sho-chū	3934 (34.4)	33.2 (31.7–34.6)
Wine	4800 (41.9)	35.4 (34.0–36.9)
Whisky	3375 (29.5)	26.7 (25.4–28.1)
Fruit wine	3103 (27.1)	24.8 (23.5–26.1)
Other liquors	1771 (15.5)	13.6 (12.5–14.6)
Alcohol-related self-injury experiencing		
Never	9755 (85.2)	86.0 (85.0–87.1)
Yes, but not in the past year	1403 (12.3)	11.6 (10.6–12.5)
Yes, in the past year	285 (2.5)	2.4 (1.9–2.9)
Alcohol-related harm to others experiencing		
Never	10,936 (95.6)	95.3 (94.7–96.0)
Yes, but not in the past year	385 (3.4)	3.4 (2.8–3.9)
Yes, in the past year	122 (1.1)	1.3 (0.9–1.7)

In addition to the past year outcomes, we also examined the proportion of participants who had ever experienced alcohol-related self-injury or harm to others not in the past year. 12.3% of participants reported experiencing alcohol-related self-injury not in the past year and 3.4% reported alcohol-related harm to others not in the past year.

### 3.3 Participants with versus without alcohol-related self-injuries within a year

Patient characteristics derived from the results of the 2025 JASTIS were further compared among the groups stratified by having experienced an alcohol-related self-injury within the previous 1 year (Table 2). An alcohol-related self-injury was experienced by 285 respondents, who were significantly younger (median, 40.0 years [IQR, 29.0–58.0] vs. 52.0 years [IQR, 39.0–65.0], *p* < 0.001) and male-dominant (68.1% vs. 59.2%, *p* = 0.003). Interestingly, the proportion of low-income respondents was significantly lower among those with alcohol-related self-injury (27.4% vs. 34.4%, *p* = 0.01), although the proportion of college graduates or higher degree holders did not differ between groups. Depression and diabetes were significantly more prevalent among those respondents with alcohol-related self-injuries.

**Table 2:** Background characteristics of participants with or without alcohol-related self-injury experiencing within a year.

	Without alcohol-related injury (n = 11,158)	With alcohol-related self-injury (*n* = 285)	*p*-value
Age, years			<0.001
15–29	1411 (12.6)	86 (30.2)	
30–39	1542 (13.8)	52 (18.2)	
40–49	2019 (18.1)	48 (16.8)	
50–59	2162 (19.4)	36 (12.6)	
60–69	2156 (19.3)	38 (13.3)	
70 and above	1868 (16.7)	25 (8.8)	
Sex, Male	6609 (59.2)	194 (68.1)	0.003
BMI, kg/m^2^			0.59
<18.5	1315 (11.8)	37 (13.0)	
18.5–25.0	7730 (69.3)	198 (69.5)	
25.0–35.0	2040 (18.3)	47 (16.5)	
35 and above	73 (0.7)	3 (1.1)	
Educational attainment, College graduate or higher degree-holder	5991 (53.7)	165 (57.9)	0.17
Low income (<400 M yen)	3840 (34.4)	78 (27.4)	0.01
Smoking status			<0.001
Never	5854 (52.5)	101 (35.4)	
Former	2881 (25.8)	83 (29.1)	
Current	2423 (21.7)	101 (35.4)	
Depression, present	414 (3.7)	35 (12.3)	<0.001
Diabetes, present	764 (6.8)	42 (14.7)	<0.001
Drinking frequency of alcohol containing drinks (AUDIT 1)			0.02
Monthly or less	2924 (26.2)	60 (21.1)	
2 to 4 times a month	2486 (22.3)	54 (18.9)	
2 to 3 times a week	2026 (18.2)	53 (18.6)	
4 or more times a week	3722 (33.4)	118 (41.4)	
Drinking amount of alcohol containing drinks (AUDIT 2)			<0.001
1 or 2 drinks	7.187 (64.4)	73 (25.6)	
3 or 4 drinks	2568 (23.0)	108 (37.9)	
5 or 6 drinks	945 (8.5)	63 (22.1)	
7, 8, or 9 drinks	274 (2.5)	23 (8.1)	
10 or more drinks	184 (1.6)	18 (6.3)	
Frequency of six or more drinks (AUDIT 3)			<0.001
Never	7224 (64.7)	61 (21.4)	
Less than monthly	2149 (19.3)	73 (25.6)	
Monthly	855 (7.7)	76 (26.7)	
Weekly	579 (5.2)	52 (18.2)	
Daily or almost daily	351 (3.1)	23 (8.1)	
Current use of each alcohol containing drinks, yes			
Beer	7375 (66.1)	175 (61.4)	0.10
Chū-hai (<1%)	2338 (21.0)	45 (15.8)	0.04
Chū-hai (3%)	3519 (31.5)	79 (27.7)	0.18
Chū-hai (4–7%)	4628 (41.5)	117 (41.1)	0.90
Chū-hai (8–9%)	2797 (25.1)	97 (34.0)	0.001
Japanese Sake	4240 (38.0)	121 (42.5)	0.14
Sho-chū	3811 (34.2)	123 (43.2)	0.002
Wine	4690 (42.0)	110 (38.6)	0.25
Whisky	3259 (29.2)	116 (40.7)	<0.001
Fruit wine	3029 (27.1)	74 (26.0)	0.69
Other liquors	1698 (15.2)	73 (25.6)	<0.001

### 3.4 Multivariable analysis of covariates including current use of each alcohol beverage on alcohol-related self-injuries within a year

**Fig. 2:**
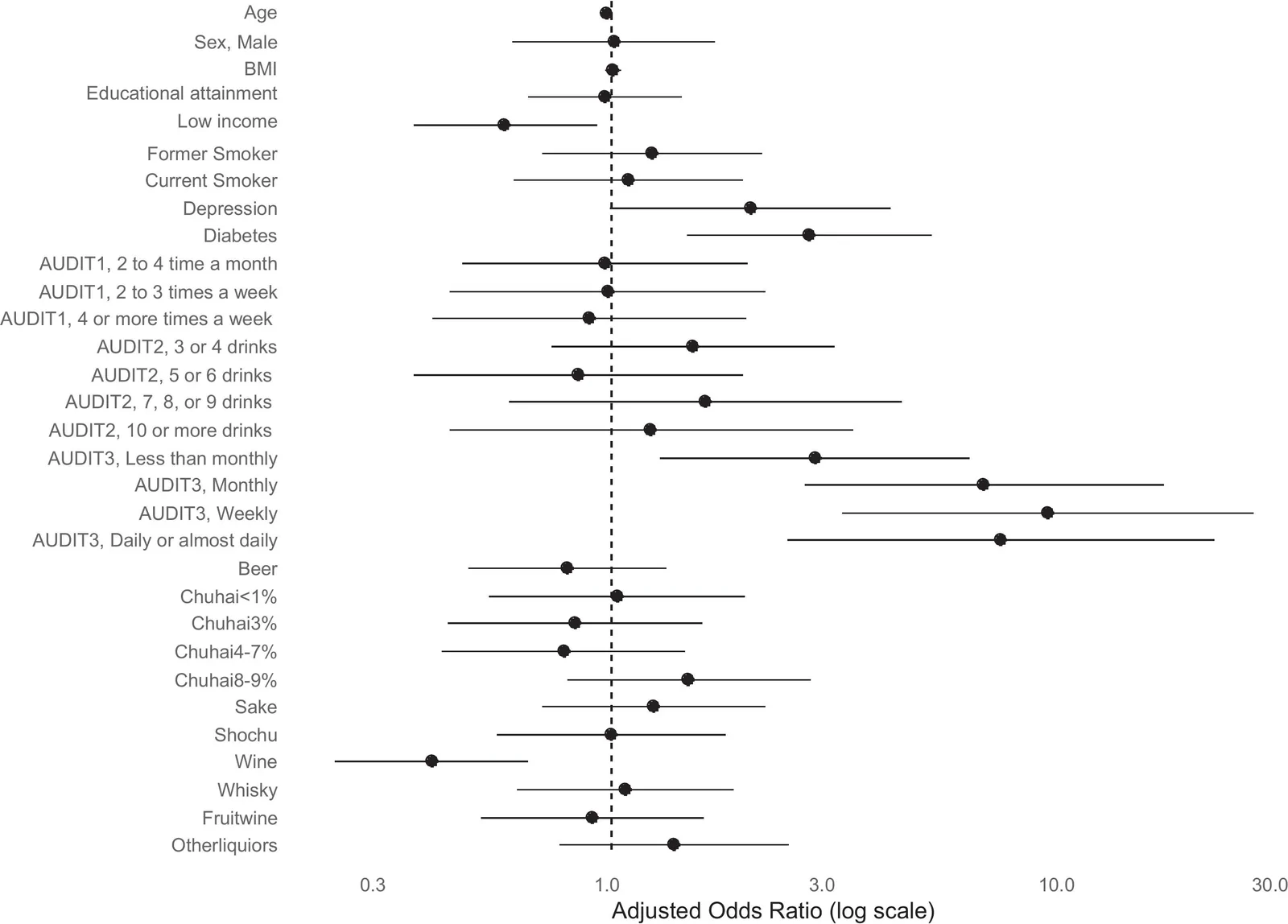
Forest plot of adjusted odds ratios of alcohol-related self-injury by beverage type.

We further performed a weighted multivariable logistic regression analysis to calculate the aOR for experiencing alcohol-related self-injuries within 1 year using variables including age, sex, BMI, educational attainment, low income, smoking status, depression, diabetes, AUDIT Q1–3, and the current use of each alcoholic beverages (Table 3, Fig. 2). In the multivariable analysis, younger individuals had a significantly higher odds of alcohol-related injury, with each additional year of age reducing the risk by about 3% (aOR per year, 0.97; 95% CI, 0.96–0.99; *p* < 0.001). Diabetes (aOR, 2.75; 95% CI, 1.46–5.16; *p* = 0.002) were significantly associated with alcohol-related self-injury. Interestingly, low household income showed a negative association (aOR, 0.58; 95% CI, 0.36–0.92; *p* = 0.02). Among the questionnaires about behavior related to alcohol drinking, a clear dose–response relationship was observed for heavy drinking episodes (≥6 drinks per occasion), with the aOR rising from 2.84 (95% CI, 1.28–6.28) for less than monthly to 9.40 (95% CI, 3.27–26.99) for weekly consumption but slightly decreased in daily or almost daily cases. Alcoholic beverage type–specific analyses showed almost marginal associations, while wine drinking showed a significant negative association to alcohol-related self-injury risk (aOR, 0.40; 95% CI, 0.24–0.65; *p* < 0.001). The event per variable was 9.2 and multicollinearity was low, with all variance inflation factors below 1.5, suggesting acceptable model stability, although some estimates may remain imprecise.

**Table 3:** Logistic regression analysis for risk factors of alcohol-related self-injury experiencing within a year.

Variable	Category	Adjusted OR	95% CI	p-value
Age (per one year)		0.97	0.96–0.99	<0.001
Sex	Female	1.00 [Reference]	–	–
	Male	1.01	0.60–1.70	0.97
BMI (per one kg/m^2^)		1.00	0.96–1.04	0.93
Educational attainment, College graduate or higher degree-holder	No	1.00 [Reference]	–	–
	Yes	0.96	0.65–1.43	0.85
Low income (<400 M yen)	No	1.00 [Reference]	–	–
	Yes	0.58	0.36–0.92	0.02
Smoking status	Never	1.00 [Reference]	–	–
	Former	1.23	0.70–2.16	0.48
	Current	1.09	0.60–1.96	0.78
Depression	No	1.00 [Reference]	–	–
	Yes	2.04	0.99–4.20	0.053
Diabetes	No	1.00 [Reference]	–	–
	Yes	2.75	1.46–5.16	0.002
Drinking frequency of alcohol containing drinks (AUDIT 1)	Monthly or less	1.00 [Reference]	–	–
	2 to 4 times a month	0.96	0.46–2.01	0.92
	2 to 3 times a week	0.98	0.44–2.19	0.95
	4 or more times a week	0.89	0.40–2.00	0.78
Drinking amount of alcohol containing drinks (AUDIT 2)	1 or 2 drinks	1.00 [Reference]	–	–
	3 or 4 drinks	1.51	0.73–3.12	0.26
	5 or 6 drinks	0.84	0.36–1.96	0.69
	7, 8, or 9 drinks	1.62	0.59–4.45	0.35
	10 or more drinks	1.22	0.43–3.46	0.70
Frequency of six or more drinks (AUDIT 3)	Never	1.00 [Reference]	–	–
	Less than monthly	2.84	1.28–6.28	0.01
	Monthly	6.75	2.68–17.01	<0.001
	Weekly	9.40	3.27–26.99	<0.001
	Daily or almost daily	7.37	2.46–22.09	<0.001
Current use of each alcohol containing drinks				
Beer	No	1.00 [Reference]	–	–
	Yes	0.79	0.48–1.32	0.37
Chū-hai (<1%)	No	1.00 [Reference]	–	–
	Yes	1.03	0.53–1.98	0.94
Chū-hai (3%)	No	1.00 [Reference]	–	–
	Yes	0.83	0.43–1.59	0.57
Chū-hai (4–7%)	No	1.00 [Reference]	–	–
	Yes	0.78	0.42–1.46	0.44
Chū-hai (8–9%)	No	1.00 [Reference]	–	–
	Yes	1.48	0.79–2.77	0.22
Japanese Sake	No	1.00 [Reference]	–	–
	Yes	1.24	0.70–2.20	0.46
Sho-chū	No	1.00 [Reference]	–	–
	Yes	0.99	0.55–1.79	0.99
Wine	No	1.00 [Reference]	–	–
	Yes	0.40	0.24–0.65	<0.001
Whisky	No	1.00 [Reference]	–	–
	Yes	1.07	0.61–1.86	0.81
Fruit wine	No	1.00 [Reference]	–	–
	Yes	0.90	0.51–1.60	0.73
Other liquors	No	1.00 [Reference]	–	–
	Yes	1.37	0.76–2.48	0.29

### 3.5 Participants with versus without alcohol-related harm to others within a year

Patient characteristics were further compared between groups stratified by having experienced alcohol-related harm to others within 1 year as derived from the 2025 JASTIS results (Table 4). Alcohol-related harm to others was reported by 122 respondents, who were significantly younger (median, 32.0 years [IQR, 27.3–40.0] vs. 52.0 years [IQR, 39.0–65.0]; p < 0.001) and male-dominant (77.0% vs. 59.3%, p < 0.001). Interestingly, the proportion of low-income individuals was also significantly lower in respondents with alcohol-related harm to others (18.0% vs. 34.4%, *p* = 0.001), although the proportion of those with college educations or higher did not differ between groups. Depression and diabetes were significantly more prevalent among respondents who reported experiencing alcohol-related harm to others.

**Table 4:** Background characteristics of participants with or without alcohol-related harm to others experiencing within a year.

	Without alcohol-related injury (n = 11,321)	With alcohol-related harm to others (*n* = 122)	p-value
Age, years			<0.001
15–29	1447 (12.8)	50 (41.0)	
30–39	1554 (13.7)	40 (32.8)	
40–49	2046 (18.1)	21 (1.72)	
50–59	2192 (19.4)	6 (4.9)	
60–69	2192 (19.4)	2 (1.6)	
70 and above	1890 (16.7)	3 (2.5)	
Sex, Male	6709 (59.3)	94 (77.0)	<0.001
BMI, kg/m^2^			0.03
<18.5	1335 (11.8)	17 (13.9)	
18.5–25.0	7845 (69.3)	83 (68.0)	
25.0–35.0	2069 (18.3)	18 (14.8)	
35 and above	72 (0.6)	4 (3.3)	
Educational attainment, College graduate or higher degree-holder	6.086 (53.8)	70 (57.4)	0.47
Low income (<400 M yen)	3896 (34.4)	22 (18.0)	0.001
Smoking status			<0.001
Never	5913 (52.2)	42 (34.4)	
Former	2941 (26.0)	23 (18.9)	
Current	2467 (21.8)	57 (46.7)	
Depression, present	421 (3.7)	28 (23.0)	<0.001
Diabetes, present	779 (6.9)	27 (22.1)	<0.001
Drinking frequency of alcohol containing drinks (AUDIT 1)			0.22
Monthly or less	2950 (26.1)	34 (27.9)	
2 to 4 times a month	2506 (22.1)	34 (27.9)	
2 to 3 times a week	2056 (18.2)	23 (18.9)	
4 or more times a week	3809 (33.6)	31 (25.4)	
Drinking amount of alcohol containing drinks (AUDIT 2)			<0.001
1 or 2 drinks	7235 (63.9)	25 (20.5)	
3 or 4 drinks	2637 (23.3)	39 (32.0)	
5 or 6 drinks	965 (8.5)	43 (35.2)	
7, 8, or 9 drinks	288 (2.5)	9 (7.4)	
10 or more drinks	196 (1.7)	6 (4.9)	
Frequency of six or more drinks (AUDIT 3)			<0.001
Never	7265 (64.2)	20 (16.4)	
Less than monthly	2192 (19.4)	30 (24.6)	
Monthly	881 (7.8)	50 (41.0)	
Weekly	613 (5.4)	18 (14.8)	
Daily or almost daily	370 (3.3)	4 (3.3)	
Current use of each alcohol containing drinks, yes			
Beer	7504 (66.3)	46 (37.7)	<0.001
Chū-hai (<1%)	2363 (20.9)	20 (16.4)	0.26
Chū-hai (3%)	3564 (31.5)	34 (27.9)	0.43
Chū-hai (4–7%)	4713 (41.6)	32 (26.2)	0.90
Chū-hai (8–9%)	2861 (25.3)	33 (27.0)	0.68
Japanese Sake	4329 (38.2)	32 (26.2)	0.006
Sho-chū	3894 (34.4)	40 (32.8)	0.77
Wine	4776 (42.2)	24 (19.7)	<0.001
Whisky	3336 (29.5)	39 (32.0)	0.55
Fruit wine	3080 (27.2)	23 (18.9)	0.04
Other liquors	1747 (15.4)	24 (19.7)	0.21

### 3.6 Multivariable analysis of covariates including current use of each alcohol beverage on alcohol-related harm to others within a year

**Fig. 3:**
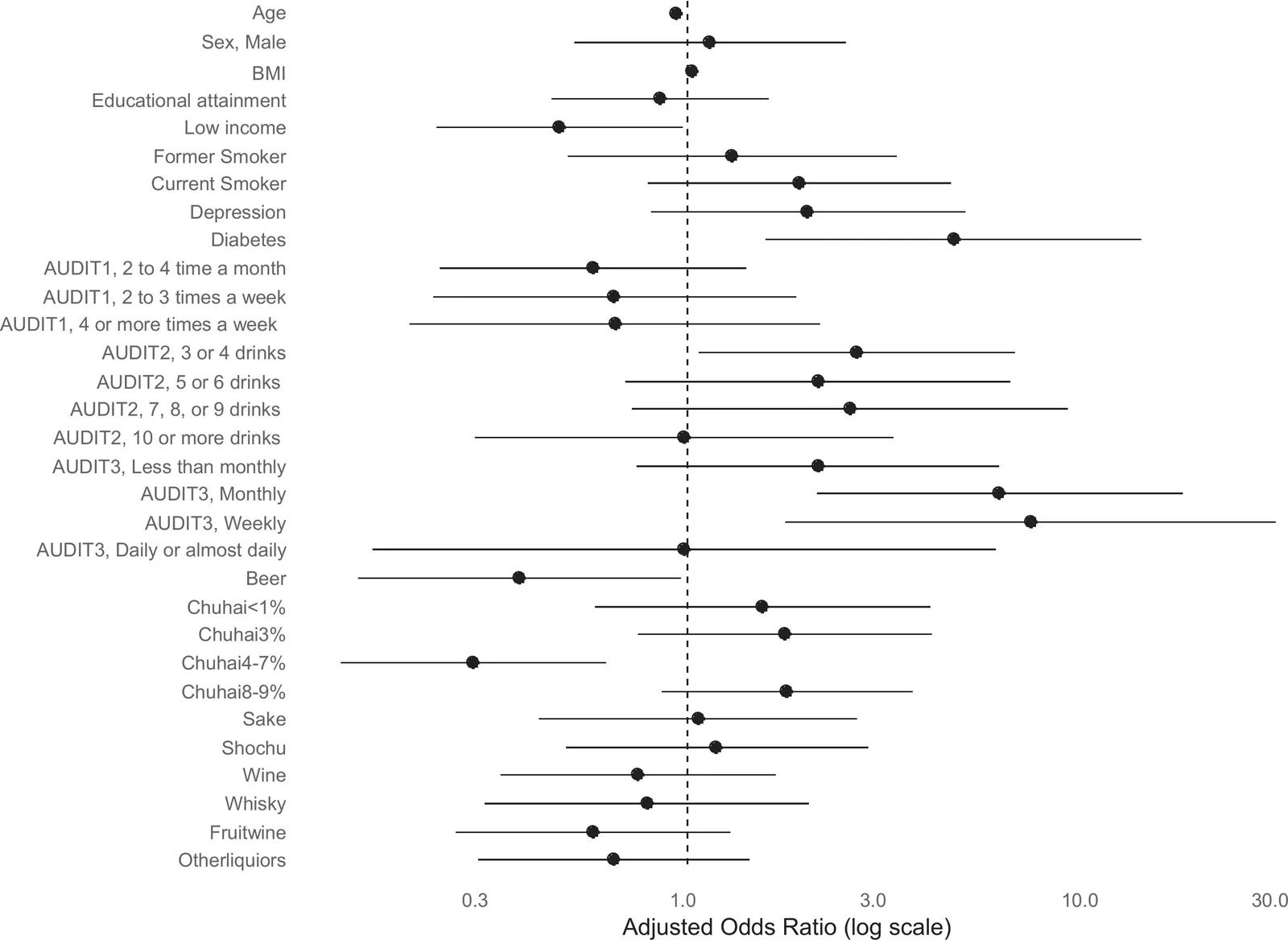
Forest plot of adjusted odds ratios of alcohol-related harm to others by beverage type.

We also performed a weighted multivariable logistic regression analysis to calculate the aOR of experiencing alcohol-related harm to others within 1 year (Table 5, Fig. 3). In this multivariable analysis, younger individuals had a significantly higher odds of alcohol-related harm to others, with each additional year of age reducing the risk by about 6% (aOR per year, 0.94; 95% CI, 0.92–0.97; p < 0.001). Diabetes was significantly associated with alcohol-related harm to others (aOR, 4.68; 95% CI, 1.58–13.9; *p* = 0.005). Interestingly, a low household income also showed a negative association (aOR, 0.48; 95% CI, 0.23–0.97; *p* = 0.04).

**Table 5:** Logistic regression analysis for risk factors of alcohol-related harm to others experiencing within a year.

Variable	Category	Adjusted OR	95% CI	p-value
Age (per one year)		0.94	0.92–0.97	<0.001
Sex	Female	1.00 [Reference]	–	–
	Male	1.14	0.52–2.50	0.75
BMI (per one kg/m^2^)		1.02	1.00–1.05	0.07
Educational attainment, College graduate or higher degree-holder	No	1.00 [Reference]	–	–
	Yes	0.86	0.46–1.61	0.63
Low income (<400 M yen)	No	1.00 [Reference]	–	–
	Yes	0.48	0.23–0.97	0.04
Smoking status	Never	1.00 [Reference]	–	–
	Former	1.29	0.50–3.36	0.60
	Current	1.91	0.79–4.61	0.15
Depression	No	1.00 [Reference]	–	–
	Yes	2.01	0.81–5.00	0.13
Diabetes	No	1.00 [Reference]	–	–
	Yes	4.68	1.58–13.9	0.005
Drinking frequency of alcohol containing drinks (AUDIT 1)	Monthly or less	1.00 [Reference]	–	–
	2 to 4 times a month	0.58	0.24–1.41	0.23
	2 to 3 times a week	0.66	0.23–1.88	0.43
	4 or more times a week	0.66	0.20–2.15	0.49
Drinking amount of alcohol containing drinks (AUDIT 2)	1 or 2 drinks	1.00 [Reference]	–	–
	3 or 4 drinks	2.67	1.07–6.67	0.04
	5 or 6 drinks	2.14	0.70–6.53	0.18
	7, 8, or 9 drinks	2.56	0.72–9.10	0.15
	10 or more drinks	0.98	0.29–3.31	0.97
Frequency of six or more drinks (AUDIT 3)	Never	1.00 [Reference]	–	–
	Less than monthly	2.13	0.74–6.12	0.16
	Monthly	6.12	2.11–17.73	0.001
	Weekly	7.31	1.77–30.25	0.001
	Daily or almost daily	0.98	0.16–5.98	0.98
Current use of each alcohol containing drinks				
Beer	No	1.00 [Reference]	–	–
	Yes	0.38	0.15–0.96	0.04
Chū-hai (<1%)	No	1.00 [Reference]	–	–
	Yes	1.55	0.59–4.09	0.38
Chū-hai (3%)	No	1.00 [Reference]	–	–
	Yes	1.76	0.75–4.13	0.19
Chū-hai (4–7%)	No	1.00 [Reference]	–	–
	Yes	0.29	0.13–0.63	0.002
Chū-hai (8–9%)	No	1.00 [Reference]	–	–
	Yes	1.78	0.86–3.68	0.12
Japanese Sake	No	1.00 [Reference]	–	–
	Yes	1.06	0.42–2.67	0.90
Sho-chū	No	1.00 [Reference]	–	–
	Yes	1.18	0.49–2.85	0.70
Wine	No	1.00 [Reference]	–	–
	Yes	0.75	0.34–1.67	0.48
Whisky	No	1.00 [Reference]	–	–
	Yes	0.79	0.31–2.03	0.63
Fruit wine	No	1.00 [Reference]	–	–
	Yes	0.58	0.26–1.29	0.18
Other liquors	No	1.00 [Reference]	–	–
	Yes	0.65	0.30–1.43	0.28

Among the questionnaires about behavior about alcohol drinking, drinking three or four drinks per day was significantly associated with alcohol-related harm to others (aOR, 2.67; 95% CI, 1.07–6.67; p = 0.04). A clear dose–response relationship was also observed for heavy drinking episodes (≥6 drinks per occasion), with the aOR rising from 2.13 (95% CI, 0.74–6.12) for less than monthly to 7.31 (95% CI, 1.77–30.25) for weekly consumption but apparently decreased in daily or almost daily cases. Interestingly, beer and *chū-hai* with a moderate ABV (4–7%) were negatively associated with the risk of alcohol-related harm to others (beer: aOR, 0.38; 95% CI, 0.15–0.96; p = 0.04; 4–7% *chū-hai*: aOR, 0.29; 95% CI, 0.13–0.63; *p* = 0.002). Overall, beverage-specific associations were less consistent associations and should be interpreted cautiously. The number of outcome events was relatively small, resulting in a low event per variable of 3.9, although multicollinearity was low, with all variance inflation factors below 1.5. Therefore, these estimates should be interpreted cautiously and considered exploratory.

## 4 Discussion

This nationwide, large-scale, and longitudinal internet survey separately examined the determinants of two types of alcohol-related injuries: self-injury and harm to others within a year. Both outcomes were associated with multiple demographic, clinical, and drinking-related factors in addition to alcohol consumption behaviors, particularly the frequency of binge drinking. Younger age and higher frequency of heavy episodic drinking showed consistent positive associations with injury outcomes. Comorbid conditions such as diabetes and depressive symptoms were also linked to higher odds of injury, whereas a low household income showed inverse associations. Beverage characteristics demonstrated heterogeneity in their relationships with outcomes: wine was negatively associated with self-injury, while beer and moderate-ABV (4–7%) RTD beverages were negatively associated with harm to others. Additionally, although statistical significance was not observed and the estimate was accompanied by substantial uncertainty, the relatively large point estimate for high-ABV (8–9%) RTD products may be hypothesis-generating. Future studies with larger sample sizes are needed to clarify whether high-ABV RTD beverages are associated with alcohol-related harm to others.

Previous epidemiological studies firmly established that alcohol-related injury risk increases in a dose–response manner with the amount of ethanol consumed and frequency of binge drinking episodes (Borges et al., 2006; Taylor et al., 2010). Our findings are in line with this evidence, showing a steep increase in injury risk with increasing frequency of consuming ≥6 drinks per occasion. The strongest association was observed in weekly binge drinking cases for both alcohol-related injury outcomes. Notably, daily or almost-daily binge drinking was not significantly associated with alcohol-related harm to others; however, this estimate was imprecise and accompanied by wide confidence intervals, and therefore should be interpreted cautiously. Some studies noted that binge alcohol consumption often occurs in home settings (Cook, MacLean, & Callinan, 2023; Holmes et al., 2024). Home drinking may allow greater freedom and reduce social monitoring, potentially facilitating increased consumption in private settings. In such contexts, opportunities for alcohol-related harm to others, such as conflict, assault, or driving, under the influence, may be relatively limited compared with those in social drinking occasions (Cook et al., 2023; Creswell, 2021). However, this interpretation remains speculative and requires further investigation.

Importantly, our findings suggest that beverage-specific associations may persist even after adjustment for measured drinking behaviors, although these findings should be considered exploratory because beverage consumption variables were not mutually exclusive and may reflect broader drinking patterns, drinking contexts, and drinker characteristics rather than independent effects of beverage type itself. Wine consumption was associated with a lower likelihood of alcohol-related self-injury, consistent with a limited number of prior studies suggesting that wine drinkers experience a lower injury risk than drinkers of other beverage types (Jani et al., 2021). Although ethanol is metabolized via the same enzymatic pathways regardless of beverage type, differences in alcohol concentration, absorption kinetics, drinking context, and accompanying non-ethanol components may contribute to the observed associations. Wine, particularly red wine, is rich in polyphenols, which have antioxidant and anti-inflammatory properties and influence vascular function. However, it remains unclear whether wine influences the kinetics of ethanol detoxification (Miraldi et al., 2024). There may be residual confounding, including overall health literacy, between wine drinkers and non-drinkers (Paschall & Lipton, 2005).

For alcohol-related harm to others, beverage-specific analyses showed negative associations of beer and moderate-ABV (4–7%) *chū-hai*, whereas high-ABV (8–9%) *chū-hai* showed a higher OR, although estimates were accompanied by substantial uncertainty. These patterns may reflect differences in alcohol concentration, speed of consumption, drinking settings, and broader drinking behaviors rather than the intrinsic properties of specific beverages. Carbon dioxide has been reported to accelerate alcohol absorption, resulting in higher peak blood alcohol concentrations (Ridout, Gould, Nunes, & Hindmarch, 2003; Roberts & Robinson, 2007), which has been shown to accelerate gastric emptying and increase the alcohol absorption rate. This may partly explain the unfavorable results of high-ABV RTD *chū-hai* (8–9%), of which ABV is even higher than beer in addition to low- or moderate-ABV *chū-hai*, although these findings should be interpreted cautiously and regarded as hypothesis-generating due to a low event per variable and limited statistical stability. These beverages may be preferentially consumed by younger and less experienced drinkers because of their attractive packaging, low cost, and ease of consumption, which could further contribute to the observed associations.

Furthermore, the associations observed with comorbid conditions suggest that an individual's underlying health status may influence vulnerability to alcohol-related injuries. We confirmed a significant risk of diabetes in alcohol-related injuries (self-injury and harm to others). Alcohol consumption by patients with diabetes, which are at higher risk of injury, particularly fractures and falls (Freire et al., 2024; Moayeri et al., 2017), has important safety concerns, especially those treated with insulin or sulfonylureas. As ethanol metabolism suppresses hepatic gluconeogenesis, alcohol consumption can precipitate hypoglycemia (Kalaria, Ko, & Issuree, 2021). Accordingly, the guidelines emphasize moderation, concurrent food intake, and patient education to reduce the alcohol-related injury burden in this vulnerable population. Our results supported these recommendations. Complications of depression also showed a marginal association with alcohol-related injuries. Depression frequently co-occurs with alcohol use disorders and has been associated with adverse behavioral outcomes, including suicidal behavior and driving under the influence (Conner et al., 2014; Y. Zhang & Sloan, 2014). These established links may partly explain the associations observed in our study and underscore the importance of considering an individual's mental health status when evaluating their alcohol-related injury risk.

An unexpected contrast is the negative association between low-income status and alcohol-related injuries. Previous studies reported that individuals in lower socioeconomic groups face a disproportionate burden of alcohol-related injuries (Zhang & Monnat, 2024). This finding suggests that younger and elderly patients, especially those with diabetes, experience alcohol-related injuries. Surprisingly, the national data in Japan presented a higher rate of traffic accidents involving individuals aged ≥50 years cycling under the influence of alcohol (Cabinet Office, 2025).

In this study, we focused on alcohol-related injuries during the previous 1 year because they more robustly reflect the relationship between continuous alcohol intake and injury outcomes. However, the overall burden of alcohol-related injury was substantially greater because a larger proportion of participants reported ever experiencing alcohol-related injury, including those who responded “yes, but not in the past year.”

Although this study provides rigorous information, it has several limitations. First, because outcomes were self-reported and involved socially sensitive behaviors, under-reporting is likely and may be related to potential under-ascertainment of alcohol-related injury outcomes (Davis, Thake, & Vilhena, 2010). In addition, restricting the analysis to continuous drinkers may have excluded individuals who reduced or stopped drinking due to prior alcohol-related harm, which may introduce selection bias and lead to underestimation of the burden of alcohol-related injury. Second, the number of outcome events was relatively small in relation to the number of covariates included in the multivariable models. The ratio of outcome events to the number of covariates may not have been sufficient to ensure stable estimates. Particularly for alcohol-related harm to others, the modest number of events may have resulted in imprecise estimates. Therefore, the beverage-specific associations should be interpreted with caution. In addition, beverage categories were not mutually exclusive, and beverage consumption variables may reflect broader drinking patterns, drinking contexts, and drinker characteristics rather than independent exposures. Residual confounding and unmeasured behavioral or social factors may partly explain the observed associations. Third, the internet-based survey design may limit the generalizability of its findings to populations with limited access or engagement with online research, although weighting was applied to approximate the general population. Finally, observational data cannot establish causality; all associations should be considered correlational.

In conclusion, alcohol-related self-injury and harm to others were mainly driven by drinking patterns and intensity, particularly heavy episodic drinking. Beverage-specific associations were weaker and should be interpreted cautiously. These findings support a multifactorial understanding of alcohol-related injury risk and alcohol harm prevention strategies.

## Declaration of generative AI and AI-assisted technologies in the manuscript preparation process

During the preparation of this work the authors used ChatGPT (version 5.2) to improve the clarity and language of the text. After using this tool, the authors reviewed and edited the content as needed and took full responsibility for the content of the published article.

## Informed consent and patient detals

Online informed consent to take part in the study and to publish the article has been obtained from all participants or their legal representatives. The privacy rights of participants have been observed.

## Studies in human

This study was performed in compliance with relevant laws, regulatory frameworks and guidelines where the research took place.

This study was approved by the Research Ethics Committee of the Faculty of Medicine, the University of Tokyo (Approval No. 2020336NI).

## CRediT authorship contribution statement

**Kazuya Okushin:** Writing – original draft, Visualization, Software, Project administration, Methodology, Investigation, Formal analysis, Conceptualization. **Takahiro Tabuchi:** Writing – review & editing, Resources, Conceptualization. **Takashi Yoshioka:** Writing – original draft, Methodology, Conceptualization. **Tomohiro Tanaka:** Writing – review & editing, Methodology, Conceptualization. **Takuma Kaneko:** Writing – review & editing. **Keisuke Mabuchi:** Writing – review & editing. **Yuki Matsushita:** Writing – review & editing. **Tomoharu Yamada:** Writing – review & editing. **Takuma Nakatsuka:** Writing – review & editing. **Tatsuya Minami:** Writing – review & editing. **Masaya Sato:** Writing – review & editing. **Yotaro Kudo:** Writing – review & editing. **Kazuhiko Koike:** Writing – review & editing, Supervision, Funding acquisition. **Mitsuhiro Fujishiro:** Writing – review & editing. **Ryosuke Tateishi:** Writing – review & editing, Supervision, Methodology, Investigation, Conceptualization.

## Declaration of competing interest

The authors declare that they have no known competing financial interests or personal relationships that could have appeared to influence the work reported in this paper.

## Data Availability

The datasets generated during the current study are not publicly available but can be provided by the corresponding author upon reasonable request.
